# Detection of spinal long fiber tract degeneration in HSP: Improved diffusion tensor imaging

**DOI:** 10.1016/j.nicl.2022.103213

**Published:** 2022-09-28

**Authors:** Tobias Lindig, Christer Ruff, Tim W. Rattay, Stephan König, Ludger Schöls, Rebecca Schüle, Thomas Nägele, Ulrike Ernemann, Uwe Klose, Benjamin Bender

**Affiliations:** a Department of Diagnostic and Interventional Neuroradiology, University Hospital Tübingen, Hoppe-Seyler-Strasse 3, Tübingen 72076, Germany; b Center for Neurology, Department of Neurodegenerative Diseases, and Hertie Institute for Clinical Brain Research, Hoppe-Seyler-Str. 3, Tübingen 72076, Germany; c German Research Center for Neurodegenerative Diseases (DZNE), Otfried-Müller-Str. 23, Tübingen 72076, Germany

**Keywords:** AH, anterior horn, AD, axial or longitudinal diffusivity, DC, dorsal columns, FA, fractional anisotropy, GM, grey matter, HSP, hereditary spastic paraplegia, sDTI, spinal diffusion tensor imaging, MD, mean diffusivity, PT, pyramidal tracts, RD, radial or perpendicular diffusivity, SPG, Spastic paraplegia, SPRS, Spastic Paraplegia Rating Scale, T, Tesla, WM, white matter, Hereditary spastic paraplegia, Pyramidal degeneration, Spinal diffusion tensor imaging, Fractional anisotropy, Radial diffusivity

## Abstract

Spinal diffusion tensor imaging (sDTI) is still a challenging technique for selectively evaluating anatomical areas like the pyramidal tracts (PT), dorsal columns (DC), and anterior horns (AH) in clinical routine and for reliably quantifying white matter anisotropy and diffusivity. In neurodegenerative diseases, the value of sDTI is promising but not yet well understood.

The objective of this prospective, single-center study was to evaluate the long fiber tract degeneration within the spinal cord in normal aging (n = 125) and to prove its applicability in pathologic conditions as in patients with molecular genetically confirmed hereditary spastic paraplegias (HSP; n = 40), a prototypical disease of the first motor neuron and in some genetic variants with affection of the dorsal columns. An optimized monopolar Stejskal-Tanner sequence for high-resolution, axial sDTI of the cervical spinal cord at 3.0 T with advanced standardized evaluation methods was developed for a robust DTI value estimation of PT, DC, and AH in both groups.

After sDTI measurement at *C*2, an automatic motion correction and an advanced semi-automatic ROI-based, standardized evaluation of white matter anisotropy and diffusivity was performed to obtain regional diffusivity measures for PT, DC, and AH. Reliable and stable sDTI values were acquired in a healthy population without significant decline between age 20 and 65. Reference values for PT, DC, and AH for fractional anisotropy (FA), mean diffusivity (MD), and radial diffusivity (RD) were established.

In HSP patients, the decline of the long spinal fiber tracts could be demonstrated by diffusivity abnormalities in the pyramidal tracts with significantly reduced PT_FA_ (p < 0.001), elevated PT_RD_ (p = 0.002) and reduced PT_MD_ (p = 0.003) compared to healthy controls. Furthermore, FA was significantly reduced in DC_FA_ (p < 0.001) with no differences in AH. In a genetically homogeneous subgroup of SPG4 patients (n = 12) with affection of the dorsal columns, DC_RD_ significantly correlated with the overall disease severity as measured by the Spastic Paraplegia Rating Scale (SPRS) (r = − 0.713, p = 0.009).

With the most extensive sDTI study in vivo to date, we showed that axial sDTI combined with motion correction and advanced data post-processing strategies enables robust measurements and is ready to use, allowing recognition and quantification of disease- and age-related changes of the PT, DC, and AH. These results may also encourage the usage of sDTI in other neurodegenerative diseases with spinal cord involvement to explore its capability as selective biomarkers.

## Highlights

• Improved spinal DTI at *C*2 allows robust column-specific measurements.
• Healthy aging shows no significant change in spinal DTI parameters up to 65 years.
• In HSP spinal tract degeneration can be reliably detected.
• In SPG4 dorsal column degeneration shows high correlation with disease severity.

## 1 Introduction

Conventional MRI has been used to visualize spinal cord disorders for many years providing excellent anatomic and morphologic information with high-resolution images. This technique is limited in detecting and quantifying the axonal degeneration of the long spinal fiber tracts, which is needed to provide outreaching information on the etiology of neurodegeneration and immediately impact clinical decision-making. Diffusion tensor imaging (DTI) is a promising imaging technology to investigate the microstructure of white matter in the spinal cord (Xu, 2013, Chan, 2015). It potentially provides quantitative measurements needed to detect spinal cord atrophy and pyramidal tract degeneration routinely but spinal DTI (sDTI) is not commonly used in radiology practice. Furthermore, existing studies often do not include a diversified age collective. Quantitative in vivo sDTI is challenging for several reasons (Clark and Werring, 2002, Barker, 2001, Maier, 2007, Mamata et al., 2005, Ellingson et al., 2007a, Rossi, 2007). The cord has a small cross-sectional area and magnetic field inhomogeneities from nearby vertebrae cause image distortions. Moreover, cerebrospinal fluid (CSF) pulsations, pulsatile pulsation in surrounding vessels, and respiratory motion generate significant motion artifacts in the anterior-posterior and rostro-caudal directions. Limited signal-to-noise ratio (SNR) resulting from physiological artifacts as well as thermal noise is a significant concern in high in-plane resolution axial sDTI acquisition. Due to these numerous reasons, no extensive in vivo cross-sectional studies have ever been conducted.

Hereditary spastic paraplegia (HSP) is an incurable degenerative motor neuron disease that primarily affects the upper motor neuron and leads to loss of ambulation. HSP is genetically heterogeneous with common clinical core symptoms: spastic gait disorder with paraparesis often accompanied by neurogenic bladder disorder. But HSP is not restricted to the motor system. It also affects the sensory system in a substantial portion of HSP patients including the long fiber tracts of the dorsal columns (Servelhere, 2021). Histopathological post-mortem analysis in HSP is rare, which often depicts the late stage of the disease (two cases with a disease duration >50 years in (Wharton, 2003). Systematic studies of MRI in representative HSP cohorts are rare and are often limited for homogenous genetic groups to the most common form with mutations in the *SPAST* gene, SPG4. MRI studies of the spinal cord are lacking but include structural cortical MRI (Lindig, 2015, Scuderi, 2009), regional cerebral blood flow analysis (Xing, 2014), diffusion tensor imaging (DTI) (Garaci, 2014), resting-state (Liao, 2018), and O^15^-positron emission tomography (PET) of the motor cortex (Scheuer, 2006).

The purpose of this study was the implementation of a high-resolution sDTI that is suitable for the selective evaluation of the pyramidal tracts (PT) and dorsal columns (DC) and detection of spinal degeneration at the cervical level where all of these long spinal tracts pass at the same level simultaneously. We hypothesized that diffusivity, especially of FA, RD, AD and MD would be significantly altered with definite illness and known pyramidal degeneration in HSP, compared to a database of normal DTI parameters gained from healthy controls (Schule, 2016, Schule and Schols, 2011, Schols, 2017). With a reference database of high-resolution DTI parameters with a broad age range, it could be possible to define the abnormality of DTI parameters even within specific spinal columns and segments. With up to 50 % sensory involvement (Schüle, 2016) (e.g. pallhypesthesia via DC), HSP as a model disease allows MRI studies with spinal cord affection beyond the pyramidal tract. Therefore, we performed a case-control study based on these two groups.

## 2 Material and methods

The local institutional ethics committee approved this single-center, cross-sectional prospective study (833/2016BO2, 115/2013BO2). Before enrollment, written informed consent was obtained from all participating HSP patients and healthy controls.

### 2.1 Study population

Recruitment of study participants with known HSP (SPG7, n = 15; SPG4, n = 12; SPG5, n = 4 and SPG11, n = 1) was coordinated through our specialized neurologic outpatient clinic. Healthy controls were taken from an in-house healthy control cohort (Lindig, 2018). Participants between the ages of 20 and 65 were included in the evaluation. Exclusion criteria were known present or past neurological and psychiatric disorders or major medical comorbidities not related to HSP, and standard contraindications for MRI. All subjects underwent a clinical neurologic examination, and HSP patients were assessed using the Spastic Paraplegia Rating Scale (SPRS) (Schüle, 2006), a reliable and valid measure of disease severity. Cognition in healthy controls was examined using the DemTect test prior to the MR scan to ensure normal cognitive abilities (Schüle, 2006, Kalbe, 2004). Data sets with strong movement artifacts were excluded after visual inspection.

### 2.2 MRI acquisition

Acquisition protocol for sDTI is provided in Supplementary Table 1. All examinations were pursued on a 3.0T MRI system (MAGNETOM Skyra, Siemens Healthineers, Erlangen, Germany) operating with a maximum gradient amplitude of 45 mT/m, a maximal slew rate of 200 mT/m/*sec*, and a 32-channel head coil. The acquisition protocol for healthy controls comprised the study protocol for sDTI and 3D brain scans with T1 and T2-FLAIR contrast according to our standard clinical protocol to screen for any pathological conditions, which were excluded. Age-related typical microangiopathy was not considered a pathological condition. MR examinations of participants with HSP included the standard clinical protocol and the above-mentioned sequences.

#### 2.2.1 Spinal DTI imaging protocol

**Supplementary figure 1:**
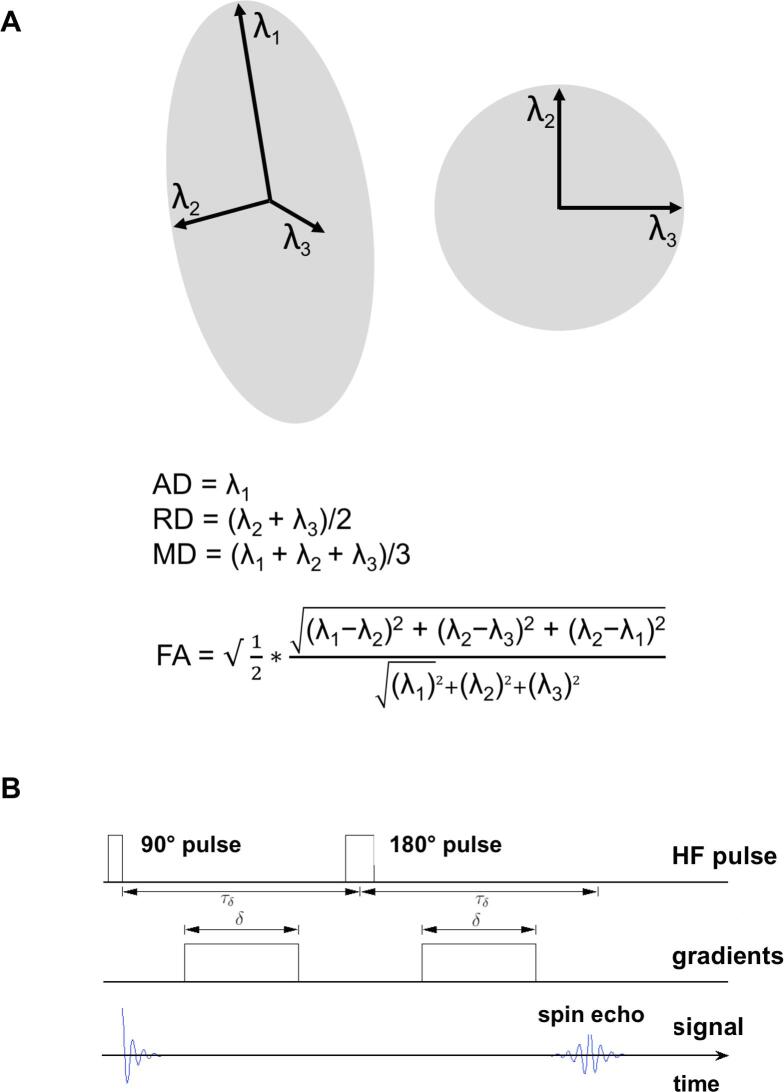


Diffusion-weighted images were acquired using an optimized monopolar, echo-planar imaging (*EPI*) sequence with double spin-echo diffusion preparation at the height of cervical vertebra two (*C*2) (Supplemental Fig. 1) (Stejskal and Tanner, 1965). At *C*2 the myelon surface area is the largest and usually a curvature is not present, yet. Because many HSP patients cannot lie still for an extended period of time, and to avoid possible slice crosstalk, an acquisition of only one slice was used. A single axial slice perpendicular to the long axis of the spinal cord was acquired to distinguish between WM and GM, and to avoid partial volume effects. Cardiac gating was applied to compensate for cerebrospinal fluid (CSF) pulsation artifacts. The best individual trigger delay was visually estimated on b0 images at the same location as the sDTI acquisition (TR 230; TE 1.46; 128×128 matrix; 5-mm section thickness; field of view of 100×100 mm^2^, in-plane resolution = 0.78×0.78 mm^2^, 5 to 8 min triggered acquisitions with individual trigger estimation). The diffusion encoding gradients of the DTI sequence were applied along the directions e1 = (1,0,1), e2 = (-1,0,1), e3 = (0,1,1), e4 = (0,1,-1), e5 = (1,1,0) and e6 = (-1,1,0). The reported coordinates (x,y,z) are relative to the scanner frame of reference; the spinal cord is parallel to the z-axis in the supine position. Two neuroradiologists evaluated diagnostic image quality with expertise in neuroanatomy on MRI scans of 13 years and 15 years. Further technical details of the sDTI sequence parameters are given in Supplemental Table 1.

#### 2.2.2 MRI post-processing and data analysis

**Fig. 1:**
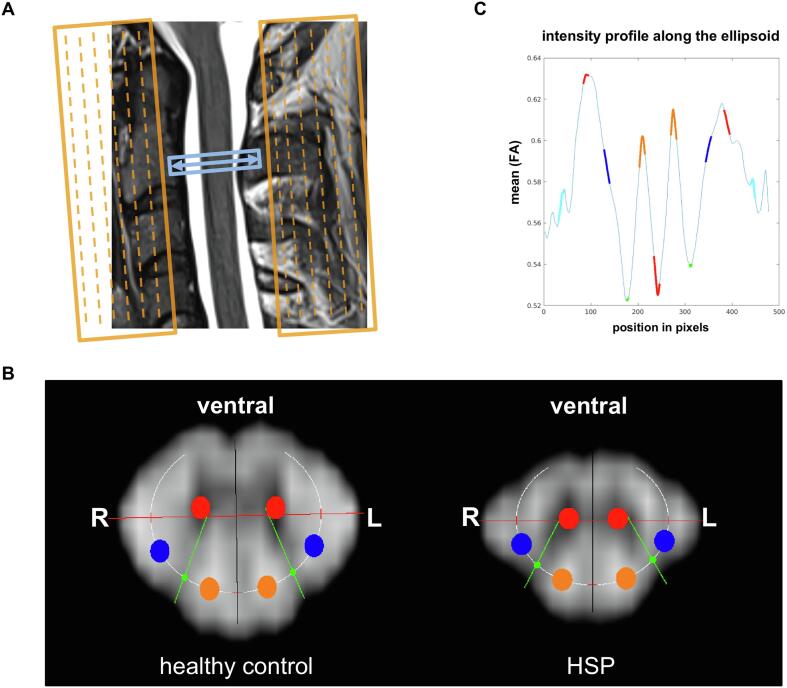
**Spinal DTI post-processing and analytic method.** (A) Acquisition of a single axial slice of 5-mm section thickness (in-plane resolution of 0.78×0.78 mm) at the second cervical vertebra. In-plane spectral fat suppression and two saturation bands along the phase encoding direction were applied to avoid folding artifacts. Phase encoding was performed along the anterior-posterior direction in the body. (B) Representative fractional anisotropy maps (FA images) of a healthy control and a study participant with HSP at the height of cervical vertebra two for semi-automated, bilateral ROI evaluation of the pyramidal tracts (PT, blue), dorsal columns (DC, orange) and anterior horns (AH, red) using an adjusted ellipsoid onto the individual FA image of each participant’s spinal cord. The long and short axis of the ellipsoid together with the exit angles of the posterior horns were adjusted in an iterative process to the plotted profile of the ellipsoid. (C) Intensity profile along the adjusted ellipsoid of the representative healthy control with respective values of PT (blue), DC (orange), AH (red) and posterior horns (green). (For interpretation of the references to colour in this figure legend, the reader is referred to the web version of this article.)

Diffusion-weighted images were analyzed offline using adapted routines written in Matlab R2015b (The MathWorks, Inc., Natick, MA). The acquired slices with the original field of view (FOV) were manually centered and cropped to the intra-spinal space at the outer margins of the cerebrospinal fluid. After exclusion of relevant eddy-current induced distortions by visual inspection, no eddy-current correction was performed. The basic diffusion measures axial diffusivity (AD) and radial diffusivity (RD) were created by fitting a tensor model to the raw diffusion data. The thereof derived diffusion measures fractional anisotropy (FA) and mean diffusivity (MD) were calculated. Since scans suffer from breathing artifacts with slice displacement in the anterior-posterior direction, an automatic movement correction was added based on midsagittal intensity profiles aligned for each direction on the b0 image. To reduce partial volume effects DTI metric maps were interpolated to 0.2×0.2 mm^2^ before placement of the ROI. To allow a semi-automated, bilateral ROI evaluation (1.05 mm in diameter) of the pyramidal tracts (PT), the dorsal columns (DC), and the anterior horns (AH), ROIs were adjusted along a manually defined ellipsoid aligned on the individual FA image of each subject’s spinal cord (Fig. 1). Both, the midline and transversal line through the most lateral aspects of the myelon as well as the gray matter that divides the DC and the lateral columns, the posterior horns, were then automatically derived by an automatic evaluation of the FA along the ellipsoid and on the transversal line (for the profile along the drawn circles, the angular sampling was 600 points for the full circle, i.e. 0.6°). The different DTI parameters were calculated as mean for each ROI accordingly. Age effects were evaluated in the healthy population. Individual DTI values of the PT, DC, and AH in HSP patients were compared to the healthy control population. For the analysis, both mean and individual values of the left and right running fiber tracts were evaluated.

### 2.3 Statistical analysis

JMP 14.2 (SAS Institute Inc., Cary, NC) and SPSS (release 26 for Windows; SPSS, Chicago, IL, USA) were used for statistical analysis. After evaluating variance between healthy controls and HSP patients with a Levene-test of equal variance, group differences were compared either with a Student’s *t*-test in case of equal variance or Welch’s *t*-test in case of unequal variance. A corrected p-value of <0.05 was considered statistically significant. Bonferroni correction was applied when multiple comparisons were calculated from the same data set. Cohen’s d effect sizes were calculated for each right and left fiber tract between HSP patients and healthy controls based on estimated marginal means (adjusted for age, age and sex), and interpreted according to the following criteria: small |d| = 0.20–0.49; medium |d| = 0.50–0.79; large |d| ≥ 0.80. Univariate GLMs with age and sex as co-founding factors and subject group (healthy control vs HSP study patient) as fixed factor were performed for in-between group differences. Linear (Pearson) correlation and quadratic regression were performed to check for a dependency of DTI measurement parameters on age, age of onset, disease duration, and SPRS.

### 2.4 Data availability

The datasets generated from the statistical analyses during the current study are available from the corresponding author on reasonable request.

## 3 Results

### 3.1 Demographics

**Supplementary figure 2:**
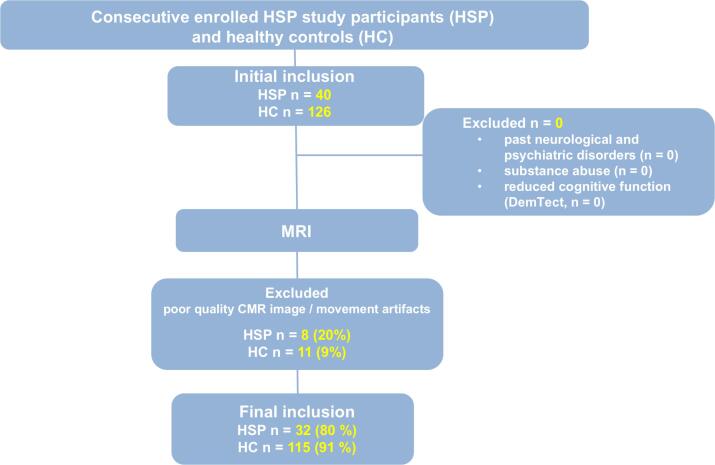


Demographic and clinical characteristics of HSP patients and healthy controls are presented in Table 1. A flowchart of the HSP study participant and healthy control selection process based on inclusion and exclusion criteria is presented in Supplemental Fig. 2. 40 patients with genetically confirmed HSP. From the 40 HSP datasets, eight scans had to be excluded due to motion artifacts or poor SNR (20 %). The HSP group consisted of 13 women (median age (IQR) 43 years (11)) and 19 men (median age (IQR) 49 years (9)). The SPRS total scores in relation to age, age of onset, and disease duration did not show any correlations between those parameters.

**Table 1:** Baseline characteristics of included study participants with HSP and healthy controls.

Parameter	HSP patients (HSP, n = 32)	healthy controls (HC, n = 115)	p value HSP vs HC
Age/years	48 (11)	37 (26)	**0.007**
Sex	13f, 19 *m*	60f, 55 *m*	Χ^2^(1, 147) = 1.336, n.s.
Age of onset/years	32 (18)		
Disease duration/years	16.5 (12)		
SPRS	17.9 ± 8.5		
sDTI MRI			
Originally performed	40	126	
Included	32 (80 %)	115 (91 %)	

f = female, m = male, SPRS = spastic paraplegia rating scale; sDTI = spinal diffusion tensor imaging. Data relating to age are presented as median and interquartile ranges (IQR). SPRS is presented as means ± standard deviation. Mann-Whitney *U* test was performed for age and Chi-square (x^2^) analysis for sex comparison. Bold are all significant p-values < 0.05.

A control group of 126 healthy participants with a similar age and sex distribution was enrolled, of which 11 scans had to be excluded due to motion artifacts (9 %). The healthy control group consisted of 60 women (median age (IQR) 36.5 years (27)) and 55 men (median age (IQR) 37 years (27)). No imaging or clinical data were suspicious for an underlying neurodegenerative process.

### 3.2 Quality control for ROI selective quantification of reliable DTI parameters of the pyramidal tracts, dorsal columns, and anterior horns

**Fig. 2:**
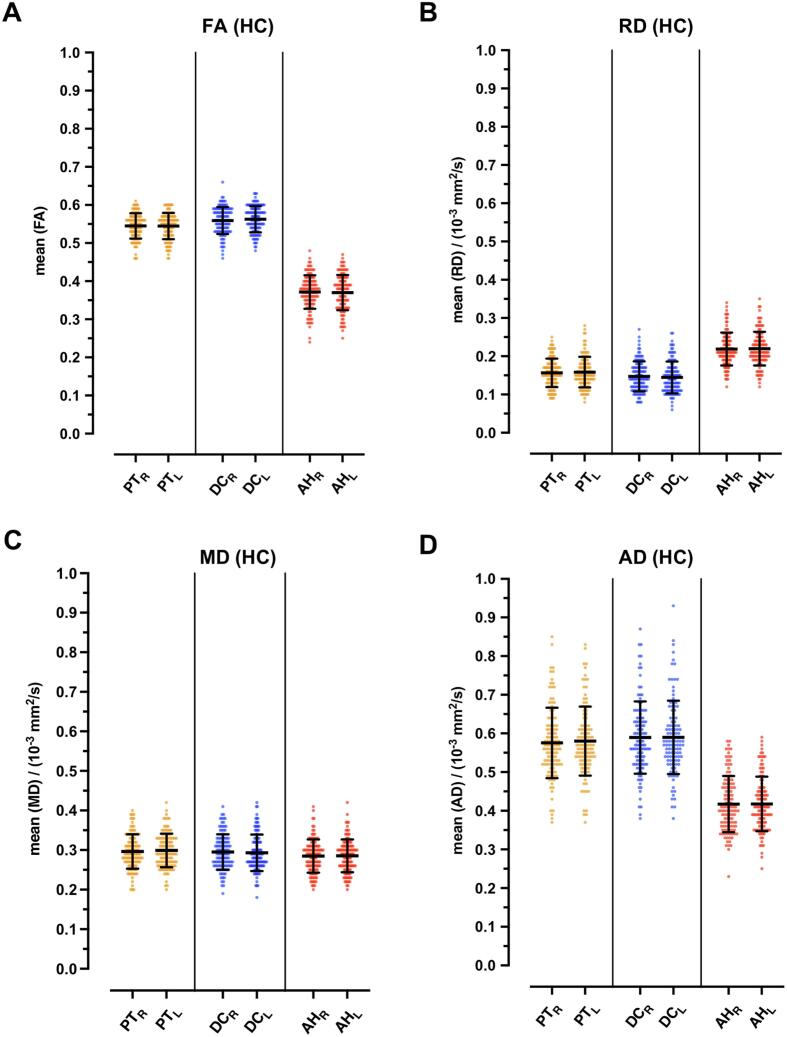
**Side-selective ROI evaluation of the diffusivity measurements of the long fiber tracts in healthy controls.** Boxplots showing comparisons of left- and right-sided (L and R in lower capitals) DTI values among healthy controls (HC, n = 115) in pyramidal tracts (PT), dorsal columns (DC) and anterior horns (AH). (**A**) Fractional anisotropy (FA); (**B**) radial diffusivity (RD); (**C**) mean diffusivity (MD); (**D**) axial diffusivity (AD). Data is shown as mean and standard deviation (SD).

Table 2 and Fig. 2 summarize quantitative measurements of anisotropy and diffusivity of healthy controls in PT, DC, and AH. Fig. 2 illustrates that no significant differences between left- and right-sided fiber tracts could be detected for all three measurement locations and the respective diffusivity parameters FA, RD, MD, and AD in healthy controls. The AD measurements show a considerably higher scattering range than FA, RD, and MD, whereas the latter show all a homogeneous distribution.

**Table 2:** Quantitative measurements of anisotropy and diffusivity in healthy controls (n = 115).

Healthy controls																				
Parameter	RD (10^−3^ mm^2^/s)					FA					MD (10^−3^ mm^2^/s)					AD (10^−3^ mm^2^/s)				
	RS	LS	p value RS vs LS	means +/- SD	span width	RS	LS	p value RS vs LS	means +/- SD	span width	RS	LS	p value RS vs LS	means +/- SD	span width	RS	LS	p value RS vs LS	means +/- SD	span width
anterior horns (AH)	0.22 ± 0.04	0.22 ± 0.04	0.856	0.22 ± 0.04	0.22	0.37 ± 0.05	0.37 ± 0.04	0.792	0.37 ± 0.04	0.23	0.29 ± 0.04	0.28 ± 0.04	0.912	0.28 ± 0.04	0.22	0.42 ± 0.07	0.42 ± 0.07	0.963	0.42 ± 0.07	0.33
dorsal columns (DC)	0.15 ± 0.04	0.15 ± 0.04	0.636	0.15 ± 0.04	0.18	0.56 ± 0.03	0.56 ± 0.04	0.439	0.56 ± 0.03	0.15	0.29 ± 0.05	0.29 ± 0.04	0.75	0.29 ± 0.03	0.23	0.58 ± 0.09	0.58 ± 0.09	0.972	0.59 ± 0.09	0.52
pyramidal tracts (PT)	0.16 ± 0.04	0.16 ± 0.04	0.733	0.16 ± 0.04	0.17	0.54 ± 0.03	0.54 ± 0.03	0.938	0.54 ± 0.03	0.15	0.30 ± 0.04	0.30 ± 0.04	0.646	0.30 ± 0.04	0.21	0.58 ± 0.09	0.58 ± 0.09	0.688	0.58 ± 0.09	0.52

Data are means +/- standard deviation (SD) with associated statistical analysis to test for significant differences. Span width is calculated of mean values. Bold are all significant p-values < 0.05. AD = axial diffusivity; FA = fractional anisotropy; LS = left side; MD = mean diffusivity; RD = radial or perpendicular diffusivity; RS = right side.

### 3.3 Evaluation of sDTI parameters in healthy controls

**Fig. 3:**
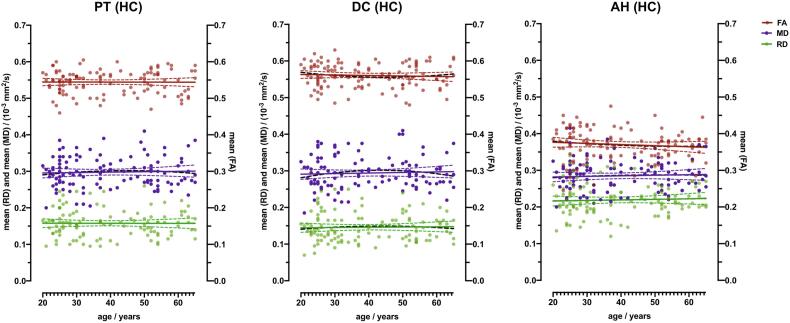
**Age distribution of fractional anisotropy (FA), mean diffusivity (MD) and radial diffusivity (RD) measured in the pyramidal tracts, dorsal columns and anterior horns of healthy controls.** ROI evaluation of FA, MD and RD in healthy controls (HC, n = 115) arranged according to age [20–65 years (y)] in pyramidal tracts (PT, (**A**)), dorsal columns (DC, (**B**)) and anterior horns (AH, (**C**)). Linear (colored) and quadratic (black dotted) regression lines are provided. 95 % CIs are presented as dotted lines.

FA, RD and MD remain stable in the healthy control group for PT, DC and AH. All three DTI parameters show no age-related changes (Fig. 3) and are stable within the age range of 20 to 65 years. Results of the diffusivity parameters are shown in Table 2. Medium effect size observed in PT for mean FA (PT_FA_) is 0.54 ± 0.03 (r(115) = − 0.005, p = 0.958), PT_RD_ 0.16 ± 0.04 10^−3^ mm^2^/s (r(115) = 0.003, p = 0.971) and PT_MD_ 0.3 ± 0.04 10^−3^ mm^2^/s (r(115) = 0.038, p = 0.69), whereas DC_FA_ is 0.56 ± 0.03 (r(115) = 0.057, p = 0.549), DC_RD_ 0.16 ± 0.04 10^−3^ mm^2^/s (r(115) = 0.023, p = 0.81) and DC_MD_ 0.30 ± 0.04 10^−3^ mm^2^/s (r(115) = 0.049, p = 0.606). Observed measurement parameters of the AH_FA_ are 0.37 ± 0.04 (r(115) = − 0.102, p = 0.282), AH_RD_ 0.22 ± 0.04 10^−3^ mm^2^/s (r(115) = 0.049, p = 0.607) and AH_MD_ 0.28 ± 0.04 10^−3^ mm^2^/s (r(115) = 0.052, p = 0.58). There were no sex related differences of FA, RD and MD in healthy controls either, except for AH_MD_ with p = 0.047 (Table 3).

**Table 3:** Results of quantitative measurements of anisotropy and diffusivity parameters of female (HC_f_, n = 60) and male healthy controls (HC_m_, n = 55) as well as quantitative measurements of diffusivity parameters of female (HSP_f_, n = 13) and male hereditary spastic paraplegia patients (HSP_m_, n = 19). Bold are all significant p-values < 0.05. FA = fractional anisotropy; MD = mean diffusivity; RD = radial or perpendicular diffusivity.

healthy controls (HC)									
parameter	RD (10^−3^ mm^2^/s)			FA			MD (10^−3^ mm^2^/s)		
	HC_m_	HC_f_	p value	HC_m_	HC_f_	p value	HC_m_	HC_f_	p value
	means +/- SD	means +/- SD		means +/- SD	means +/- SD		means +/- SD	means +/- SD	
anterior horns (AH)	0.22 ± 0.04	0.22 ± 0.04	0.343	0.56 ± 0.03	0.56 ± 0.03	0.714	0.29 ± 0.04	0.28 ± 0.04	0.047
dorsal columns (DC)	0.14 ± 0.08	0.15 ± 0.1	0.695	0.54 ± 0.03	0.55 ± 0.03	0.110	0.29 ± 0.08	0.3 ± 0.05	0.677
pyramidal tracts (PT)	0.16 ± 0.04	0.15 ± 0.04	0.216	0.37 ± 0.04	0.37 ± 0.37	0.637	0.30 ± 0.04	0.29 ± 0.05	0.254
HSP patients (HSP)									

parameter	RD (10^−3^ mm^2^/s)			FA			MD (10^−3^ mm^2^/s)		
	HSP_m_	HSP_f_	p value	HSP_m_	HSP_f_	p value	HSP_m_	HSP_f_	p value
	means +/- SD	means +/- SD		means +/- SD	means +/- SD		means +/- SD	means +/- SD	
anterior horns (AH)	0.21 ± 0.04	0.20 ± 0.35	0.635	0.37 ± 0.05	0.38 ± 0.04	0.444	0.29 ± 0.03	0.28 ± 0.03	0.280
dorsal columns (DC)	0.15 ± 0.03	0.16 ± 0.04	0.701	0.53 ± 0.04	0.52 ± 0.05	0.597	0.31 ± 0.03	0.31 ± 0.04	0.823
pyramidal tracts (PT)	0.18 ± 0.04	0.18 ± 0.03	0.812	0.49 ± 0.04	0.49 ± 0.03	0.937	0.33 ± 0.04	0.31 ± 0.03	0.233

**Supplementary figure 3:**
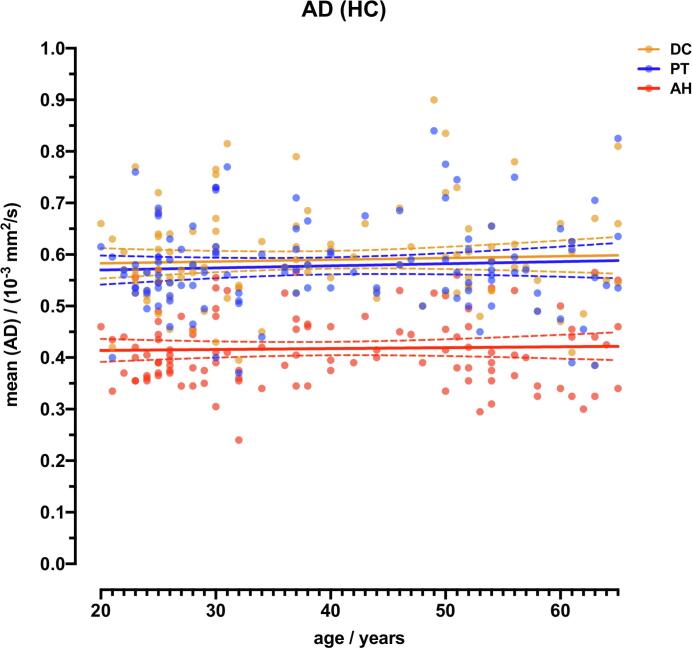


Graphical representation of AD data with a higher scattering range is shown in Supplemental Fig. 3 for completeness. In contrast to the other diffusivity parameters, the present model with axial DTI measurements of a single slice seems not to be suitable to provide valid values for AD, i.e. diffusivity craniocaudal and caudocranial, as can be seen from the widespread distribution of measured values in HC (mean PT_AD_ 0.58 ± 0.09 10^−3^ mm^2^/s, span width PT 0.52; mean DC_AD_ 0.59 ± 0.09 10^−3^ mm^2^/s, span width DC 0.52; mean AH_AD_ 0.42 ± 0.07 10^−3^ mm^2^/s, span width 0.33) in contrast to the relatively closely packed values of FA, RD and MD (Fig. 3, Table 2). Despite the widespread distribution of AD measurements, linear regression shows no significant changes for PT (r(115) = 0.064, p = 0.497), DC (r(115) = 0.053, p = 0.576) and AH (r(115) = 0.035, p = 0.707), Supplemental Fig. 3. Since axial sDTI does not seem to achieve valid values for AD, no further statistical calculation between healthy controls and HSP patients were performed.

### 3.4 Evaluation of sDTI parameters in HSP patients

**Fig. 4:**
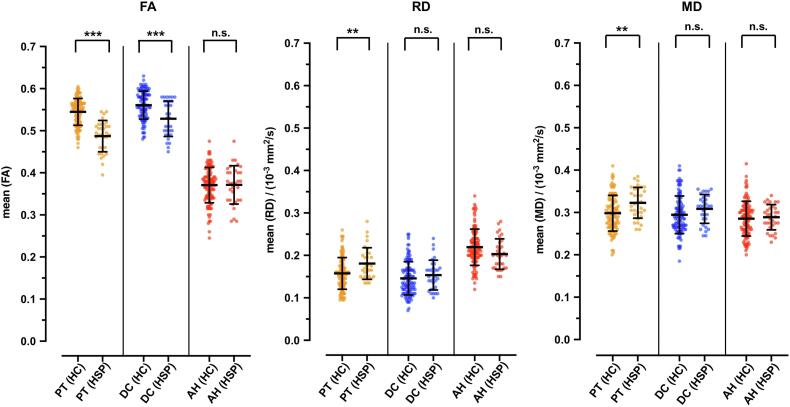
**Differences of fractional anisotropy (FA), radial diffusivity (RD) and mean diffusivity (MD) between healthy controls and HSP patients.** Boxplots showing between group-differences of FA, RD and MD among healthy controls (HC, n = 115) and HSP study participants (HSP, n = 32) age independent in pyramidal tracts (PT), dorsal columns (DC) and anterior horns (AH). See main text for further details. Significant differences are indicated with asterisks (* p < 0.05, ** p < 0.01, *** p < 0.001, n.s. = not significant). Data is shown as mean and standard deviation (SD).

DTI parameters including FA, RD and MD in the three investigated fiber tract regions PT, DC and AH were compared between healthy controls and HSP patients (Table 4, Fig. 4). There is a significant reduction of FA in the PT (0.54 ± 0.03 vs 0.49 ± 0.04, p < 0.001) and the DC_FA_ (0.56 ± 0.03 vs 0.52 ± 0.04, p < 0.001) in patients with HSP compared to healthy controls.

**Table 4:** Results of quantitative measurements of anisotropy and diffusivity of HSP patients (n = 32) and healthy controls (n = 115).

Parameter	RD (10^−3^ mm^2^/s)				FA				MD (10^−3^ mm^2^/s)			
	HSP	HC	p value	Cohen’s D	HSP	HC	p value	Cohen’s D	HSP	HC	p value	Cohen’s D
	means +/- SD	means +/- SD			means +/- SD	means +/- SD			means +/- SD	means +/- SD		
anterior horns (AH)	0.20 ± 0.04	0.22 ± 0.04	0.052	−0.391	0.37 ± 0.05	0.37 ± 0.04	0.937	0.016	0.29 ± 0.03	0.28 ± 0.04	0.614	0.609
dorsal columns (DC)	0.15 ± 0.04	0.15 ± 0.04	0.323	0.198	0.52 ± 0.04	0.56 ± 0.03	**< 0.001**	−0.965	0.31 ± 0.03	0.29 ± 0.04	0.92	0.339
pyramidal tracts (PT)	0.18 ± 0.04	0.16 ± 0.04	**0.002**	0.617	0.49 ± 0.04	0.54 ± 0.03	**< 0.001**	−1.731	0.32 ± 0.04	0.30 ± 0.04	**0.003**	0.101

Data are means +/- standard deviation (SD) with associated statistical analysis to test for significant differences. *P* value is for all study participants versus healthy participants. Bold are all significant p-values < 0.05. FA = fractional anisotropy; HC = healthy controls; MD = mean diffusivity; RD = radial or perpendicular diffusivity; HSP = hereditary spastic paraplegia patients.

Furthermore, RD is significantly increased in the PT (0.16 ± 0.04 vs 0.18 ± 0.04 10^−3^ mm^2^/s, p = 0.002) whereas there is no significant difference in the DC_RD_ (0.15 ± 0.04 vs 0.15 ± 0.04 10^−3^ mm^2^/s, p < 0.198). Significant differences could also be detected for MD in the PT (0.32 ± 0.04 vs 0.30 ± 0.04 10^−3^ mm^2^/s, p = 0.003) but not in DC_MD_ (0.31 ± 0.03 vs 0.29 ± 0.04 10^−3^ mm^2^/s, p = 0.92).

No significant change of mean FA (0.37 ± 0.04 vs 0.36 ± 0.05, p < 0.937), RD (0.20 ± 0.04 vs 0.22 ± 0.04 10^−3^ mm^2^/s, p < 0.052) and MD (0.29 ± 0.03 vs 0.28 ± 0.04 10^−3^ mm^2^/s, p < 0.614) could be observed in AH.

Table 3 provides, in addition, the gender comparison for the diffusivity parameters of HSP patients. No significant gender specific differences could be detected for FA, RD and MD in the PT, DC and AH. In concordance with these results an additional GLM that took age and gender as co-founding factors found only group comparison as a significant influencing factor for the above mentioned tracts (see Supplemental Table 2).

### 3.5 Spinal DTI parameters association with clinical parameters

**Fig. 5:**
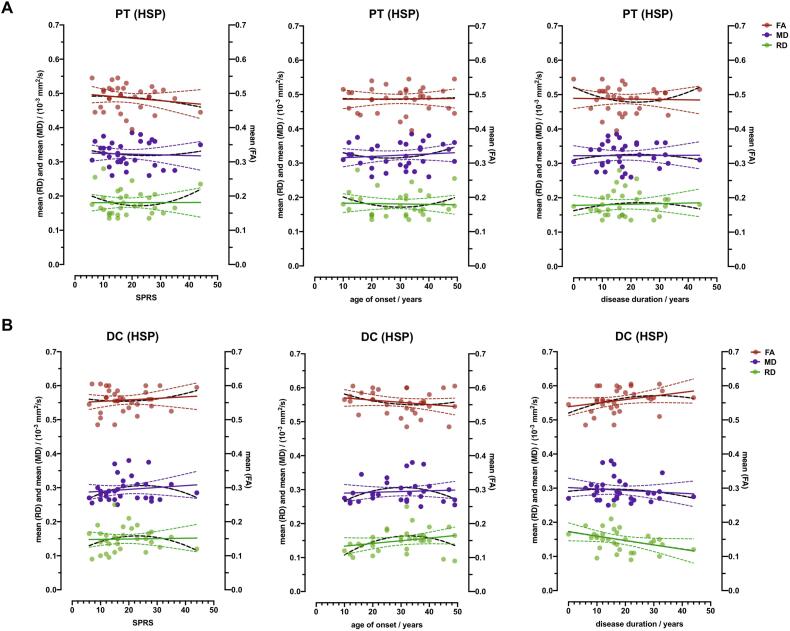
**All HSP study participants. Correlation analysis of fractional anisotropy (FA), mean diffusivity (MD) and radial diffusivity (RD) compared to the Spastic Paraplegia Rating Scale score (SPRS), age of onset and disease duration.** ROI evaluation of FA, MD and RD of all HSP study participants (HSP, n = 32) in the pyramidal tracts (PT) and dorsal columns (DC) arranged according to SPRS, age of onset (in years (y)) and disease duration. Linear regression lines are provided. 95 % CIs are presented as dotted lines.

No significant correlation between the Spastic Paraplegia Rating Score (SPRS) and the DTI parameters FA, RD and MD could be shown in pyramidal tracts and dorsal columns across all study participants (Fig. 5). In HSP patients, linear correlation analysis did not show a significantly non-zero slope for FA in DC (r(32) = 0.114, p = 0.534) and PT_FA_ (r(32) = − 0.169, p = 0.354), for RD in DC (r(32) = 0.027, p = 0.883) and PT_RD_ (r(32) = 0.003, p = 0.984) as well as for MD in DC (r(32) = 0.139, p = 0.447) and PT_MD_ (r(32) = − 0.05, p = 0.787). Furthermore, no significant correlation could be detected between these DTI parameters neither with age of onset nor disease duration.

**Fig. 6:**
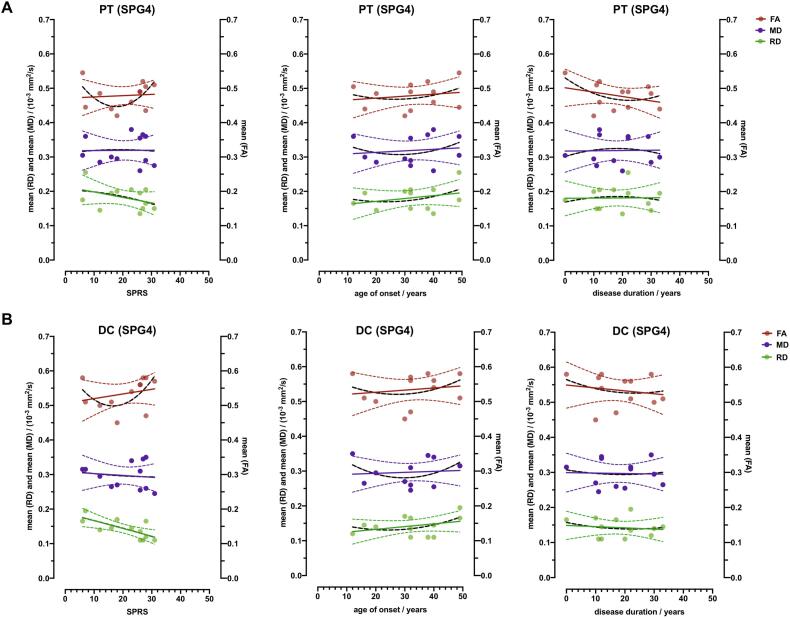
**HSP SPG4 subgroup. Correlation analysis of fractional anisotropy (FA), radial diffusivity (RD) and mean diffusivity (MD) compared to the Spastic Paraplegia Rating Scale score (SPRS), age of onset and disease duration.** ROI evaluation of FA, MD and RD of the SPG4 subgroup (HSP, n = 12) in the pyramidal tracts (PT) and dorsal columns (DC) arranged according to SPRS, age of onset (in years (y)) and disease duration. Linear regression lines are provided. 95 % CIs are presented as dotted lines.

To check whether the homogeneous SPG4 subgroup shows different results than a heterogenous group of HSP patients, diffusion parameters were evaluated for SPG4 (n = 12, Fig. 6) patients only and correlated with the SPRS score, age of onset and disease duration. However, these also showed no significant correlations except for DC_RD_ with good correlation to the SPRS score in SPG4 (r(12) = − 0.713, p = 0.009). No significant correlations could be detected for PT_RD_ of the SPG4 subgroup (r(12) = − 0.397, p = 0.201).

## 4 Discussion

HSP is the prototype disease of a continuous and slow degeneration of the first motor neuron not detectable by conventional MRI. In this study, the value of an improved sDTI and its quantitative parameters could be demonstrated by confirming spinal pyramidal and dorsal column degeneration in HSP patients. A significant decline in fractional anisotropy and mean diffusivity together with corresponding increase in radial diffusivity could be shown in the pyramidal tracts compared to healthy controls. Moreover, improved sDTI enables the selective evaluation not only of the pyramidal tracts but also of the dorsal columns and the anterior horns in line with high prevalence of sensory and low prevalence of second motor neuron involvement in HSP especially in the majority of cases included in this study (Schüle, 2016).

### 4.1 Biological basis of the altered diffusivity

The biological basis of these specific changes can be explained by neuropathological post mortem studies in HSP. These studies report axon degeneration involving lateral corticospinal tracts that is less severe in the cervical spinal cord and most severe in the thoracic spinal cord (at the distal ends) ((Wharton, 2003, Fink, 2013). In addition to distal corticospinal tract degeneration, degeneration of axons of the fasciculus gracilis fibers is consistently observed, running from caudal to cranial and is most pronounced in the cervical spinal cord (Wharton, 2003, Fink, 2013). In line with this, we could demonstrate a good and significant correlation of RD with the overall disease severity SPRS score for selective DTI measurements of the long fiber tracts in the dorsal columns of the upper cervical spinal cord. Consequently, in future studies, recordings in the inferior thoracic spinal cord are necessary to detect the full extent of pyramidal tract degeneration and thus show a correlation with the clinical severity of motor symptoms. Axon degeneration may cause mild to marked atrophy of the spinal cord in the cervical and thoracic segments. Demyelination of degenerating corticospinal tracts and fasciculus gracilis fibers is noted frequently and generally considered to indicate the degree of axon degeneration rather than signifying a primary demyelinating process (Fink, 2013). This is in line with the demonstrated alterations of the DTI values FA, MD and RD in the pyramidal tracts and dorsal columns, the latter contain ascending sensory pathways. In general, SPG5 and SPG4 are considered to be pure HSPs with regards to Anita Harding’s classification (Schols, 2017, Parodi, 2018, Harding, 1983). For both genetic subtypes, lower motor neuron (LMN) involvement is rather rare according to literature. SPG7 itself is considered to be a complicated form presenting with cerebellar ataxia. In SPG7, there are studies describing a lower motor neuropathy. The frequency is variable between 0 % reported by Karle et al. up to approximately 30 % of cases according to van Gassen (Karle, 2013, van Gassen, 2012). The largest study with about 240 affected SPG7 patients reported muscle wasting as indirect sign for motor neuropathy in 15–17 % of cases with electrophysiological measurements lacking (Coarelli, 2019). With only one SPG11 case included in this study, there won’t be a dominant LMN involvement. Accordingly, the DTI parameters FA, MD and RD of the anterior horns show no significant difference between HSP patients and healthy controls.

### 4.2 Improvements of sDTI in healthy controls

The findings of this study are an important advancement beyond conventional MRI and previous studies of sDTI. The results of this study highlight the importance of column-specific analysis in applying DTI in evaluation of spinal cord disorders. For the most part, previous studies lack both the size of the collective comparable to ours and a high-resolution representation of the myelon. This study revealed no significant age-related changes in standard DTI parameters in the healthy control group. Previous sDTI studies show different results regarding age dependency (Chan, 2015, Chagawa, 2015). One study, including 96 healthy controls in the age range of 13 to 70 years (mean 43 years) found significant age-related changes of the averaged fractional anisotropy of the cervical spinal cord, but not for mean diffusivity or cross-sectional area (Agosta, 2007). Another study including 36 healthy control age range 20–77 years (mean 51.3 years) also showed age-related changes of FA (Wang, 2014). The age range in these studies was slightly larger, and a decrease in FA in other studies was especially seen in elderly which might explain the missing age-dependence in our cohort. Furthermore, only a whole cord analysis from sagittal images or analysis on images with low axial in-plane resolution without motion correction and with freehand ROI placement were performed in these studies.

Multiple technical factors influence the quality of sDTI. The sDTI sequence used in this study with a low FOV, high axial in-plane resolution and motion correction represents a strong advance compared to other studies which frequently use sagittal acquisitions with low axial resolution and freehand-ROI placement onto an axial fractional anisotropy map for whole cord DTI metrics or estimated location of spinal tracts which might lead to incorrect results (Wang, 2014, Brander, 2014, Crombe, 2016). Data processing, especially motion correction and spinal cord segmentation are a necessary step towards robust and automated interpretation of morphometric and multiparametric MR images (Xu, 2013). In this study, a semi-automatic approach was utilized with a novel bilateral, geometry-based ROI approach allowing for a more unbiased segmentation of white and gray matter i.e. pyramidal tracts, dorsal columns and the anterior horns. The results of this study show that this evaluation method for sDTI is technically functioning and reliable for regionally selective FA, RD, and MD measurements. Another similar semi-automatic approach was used and evaluated by Xu et al. and Klawiter et al. before (Xu, 2013, Klawiter, 2012). The geometry-based ROI segmentation techniques have been shown to have advantages over tractography or fuzzy-logic approaches (Ciccarelli, 2008, Ellingson et al., 2007b).

### 4.3 Clinical impact and implication of sDTI

An imaging method that can objectively quantify the neurodegeneration of the long fiber tracts is greatly needed for the central nervous system. So far it has not been possible to evaluate the entire spectrum and severity of pyramidal degeneration on the basis of other previous imaging techniques or electrophysiology which allows only a graduation of minor damage whereas advanced stages can no longer be evaluated. When the motor evoked potentials (MEP’s) have already ceased, to a stage of the disease in which patients can still walk, the further progression of the disease can no longer be monitored with electrophysiology (Karle, 2013). A recent study by Servelhere et al. found a statistically significant volume reduction of the cervical spine at level *C*2 – C4 in patients compared to healthy controls with SPG4 and SPG11 but not for patients with SPG7. An association of the duration and severity of clinical disability with reduced gray matter area could only be shown at the level of C4 (Servelhere, 2021). Changes in sDTI parameters might precede volume reduction and are a sign of altered microstructure, even before volume loss and therefore sDTI might bridge this gap and opens a wide range of possibilities in clinical and scientific work regarding monitoring disease progression, treatment response and guidance of therapeutic decisions throughout the entire course of the disease.

This study shows, using the homogeneous SPG4 subgroup, that there is a correlation of RD from the dorsal columns to disease severity as measured by the SPRS score. It must be noted, however, that there were nearly twice as many movement artifacts in the study group with HSP as in the comparison group. This can be explained by the onset of spasticity during the measurements, which makes it difficult for patients with this condition to lie still permanently. The results of this study highlight the importance of column-specific analysis in applying DTI in evaluation of spinal cord disorders. With DTI analysis only as in a whole cord region, details within each white matter column cannot be sufficiently revealed. In a whole cord analysis, subtle abnormalities of DTI parameters caused by minor microstructure impairment in a small region may be missed. A recent study by Navas-Sanchez et al. evaluated the SPG4 subset of 12 patients and found FA changes which were significantly related to disease severity as measured by SPRS (Navas-Sanchez, 2022). The data presented here did not show a significant correlation between FA and disease severity but from Fig. 4 it becomes obvious this might be caused by two potential outliers, and missing correlation might be caused just by the sample size. Both, the current study and the study by Navas-Sanchez et al. only included a small number of SPG4 patients and further studies need to verify the changes, and need to evaluate DTI changes in a longitudinal design. One limitation of the study by Navas-Sanchez et al. is the sagittal acquisition of the DTI parameters that comes with secondary axial reconstructions. The presented method here allows for a higher in-plane resolution, which in theory should decrease partial volume effects. A further improvement can probably be achieved with new acquisition techniques like selective volume imaging (e.g. ZOOMit) that allow for a faster acquisition, an even higher resolution, with less susceptibility artifacts and distortions.

### 4.4 Limitations

This study has several limitations. First, with HSP being a rare disease the access to affected patients is limited. Second, a longitudinal study for a correlation of DTI parameters to individual changes in clinical severity of the disease measured by the Spastic Paraplegia Rating Scale (SPRS) is still lacking. Third, the number of diffusion directions are important for fiber tracking and advanced diffusion parameters. In this study only 6 directions were used as the basic parameters (FA, MD, RD) have been shown to be similar independent of the number of diffusion directions for different tracts (Lebel et al., 2012). Nonetheless, higher number of diffusion directions could decrease technical induced variability of the results. Fourth, the general reproducibility of this study using 3.0T scanners from different vendors and also for allowing the quantitative comparison of DTI values have not been assessed and needs to be evaluated in the future. Therefore, future larger prospective studies even in multi-center approaches with various scanners are needed to further validate our data, prove the general reproducibility and if necessary to introduce scanner dependent cut-off values between normal and pathologic conditions. Another possible improvement would be the transfer from semi-automatic motion correction used in this study to a fully automatic motion correction and spinal cord segmentation; allowing an even better clinical usability and reduction of possible rater bias. The introduction of sophisticated and advanced acquisition techniques as compressed sensing have shown to be able to accelerate DTI and might improve clinical use as well (Shi, 2015, Wu, 2014). Nevertheless, the study results point to a potential path toward a better clinical usability of sDTI and distinction between normal and pathological conditions.

### 4.5 Conclusion

In conclusion, the results of this study demonstrate a pattern of robust alterations of pyramidal tracts and dorsal columns within HSP and reveal unique features including the distinction of normal ageing and pathologic conditions of the myelon compared to a healthy control group using an improved sDTI sequence. Therefore, it could have an important impact on patient management from diagnosis to therapy. As HSP is the prototype disease of the degenerating first motor neuron, the measured increase of radial diffusivity and decline of fractional anisotropy and mean diffusivity in the pyramidal tracts reflect well this pathologic condition. These patterns may help to detect a measurable correlate in patients with suspected motoneuron disease as well as the extent of microstructural changes, degeneration and outcome.

An ideal future study would further investigate sDTI as a graduation and prognostic tool for further disease development and evaluation of response to therapy in myelon diseases affecting the long fiber tracts such as HSP, amyothrophic lateral sclerosis, Friedreich ataxia, spinal cord ischemia, spinal tumors or multiple sclerosis.

**Competing interests**.

BB is co-founder and share-holder of AIRAmed and has received consultancy fees from Medtronic; TL is co-founder, CEO and share-holder of AIRAmed, he has received grants from the Alzheimer Forschung Initiative e.V., travel grants from Bayer and presentation fees from Roche. The other authors report no competing interests.

## Declaration of Competing Interest

The authors declare that they have no known competing financial interests or personal relationships that could have appeared to influence the work reported in this paper.

## Data Availability

Data will be made available on request.
