# Supplementary material for: Detection of spinal long fiber tract degeneration in HSP: Improved diffusion tensor imaging
Source: Neuroimage Clin. 2022 Sep 28;36:103213. doi: 10.1016/j.nicl.2022.103213 (PMC9668628; doi:10.1016/j.nicl.2022.103213)
Supplement: Supplementary data 1 [file mmc1.docx]

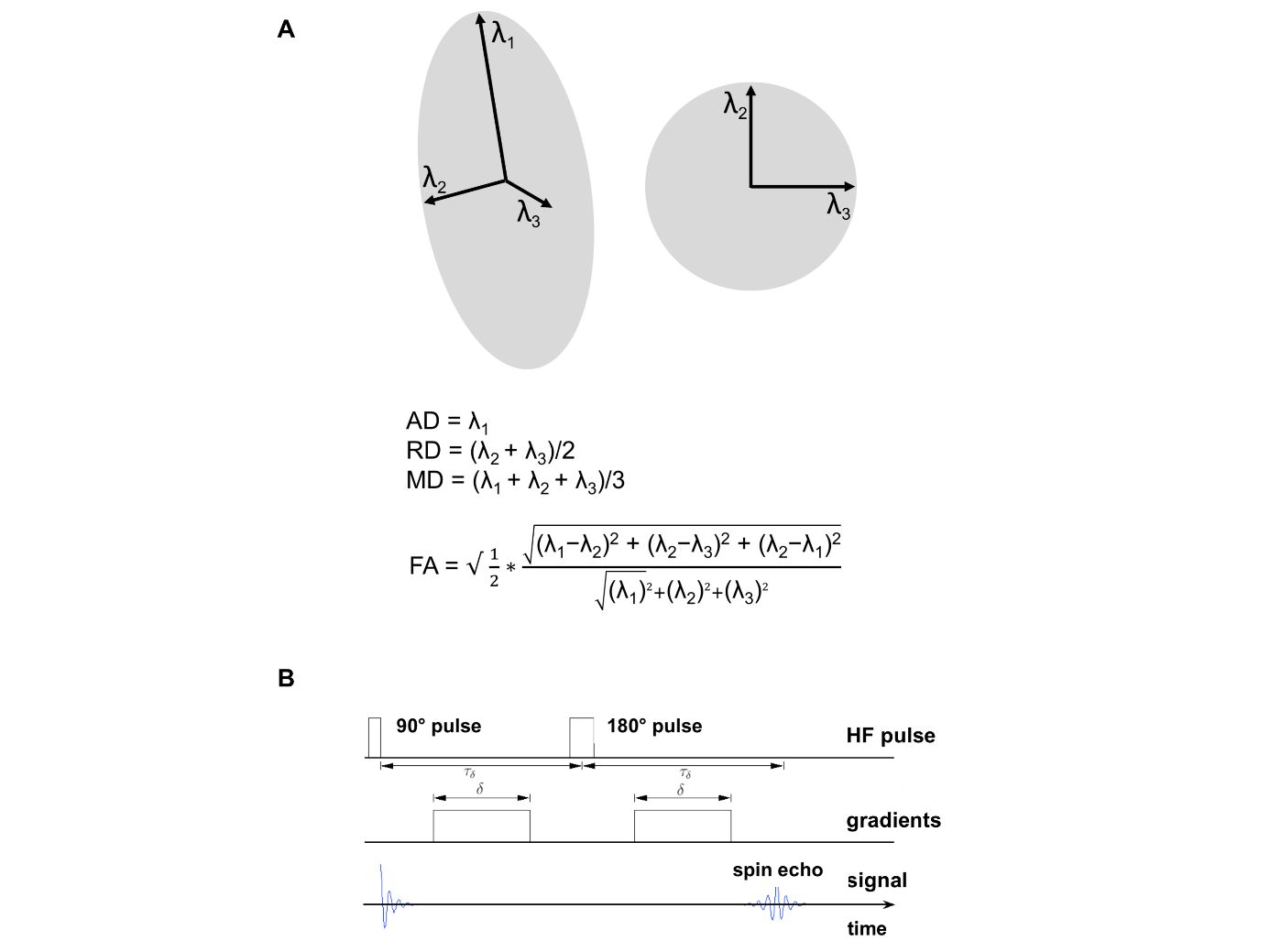


**Supplemental Figure 1: Acquisition scheme of sDTI and schematic representation of diffusor tensor imaging (DTI) derived values.**


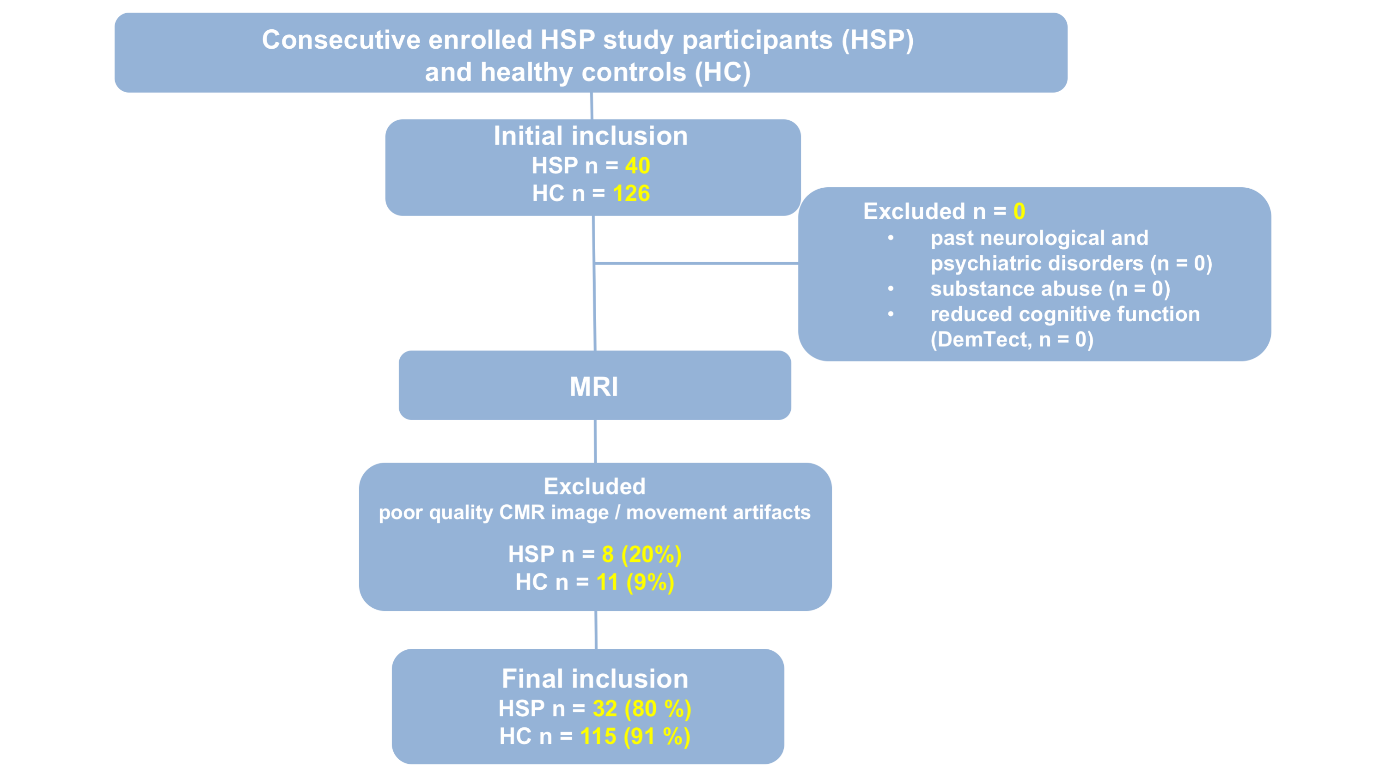


**Supplemental Figure 2:** **Flowchart shows HSP study participant and healthy control selection process based on inclusion and exclusion criteria.**


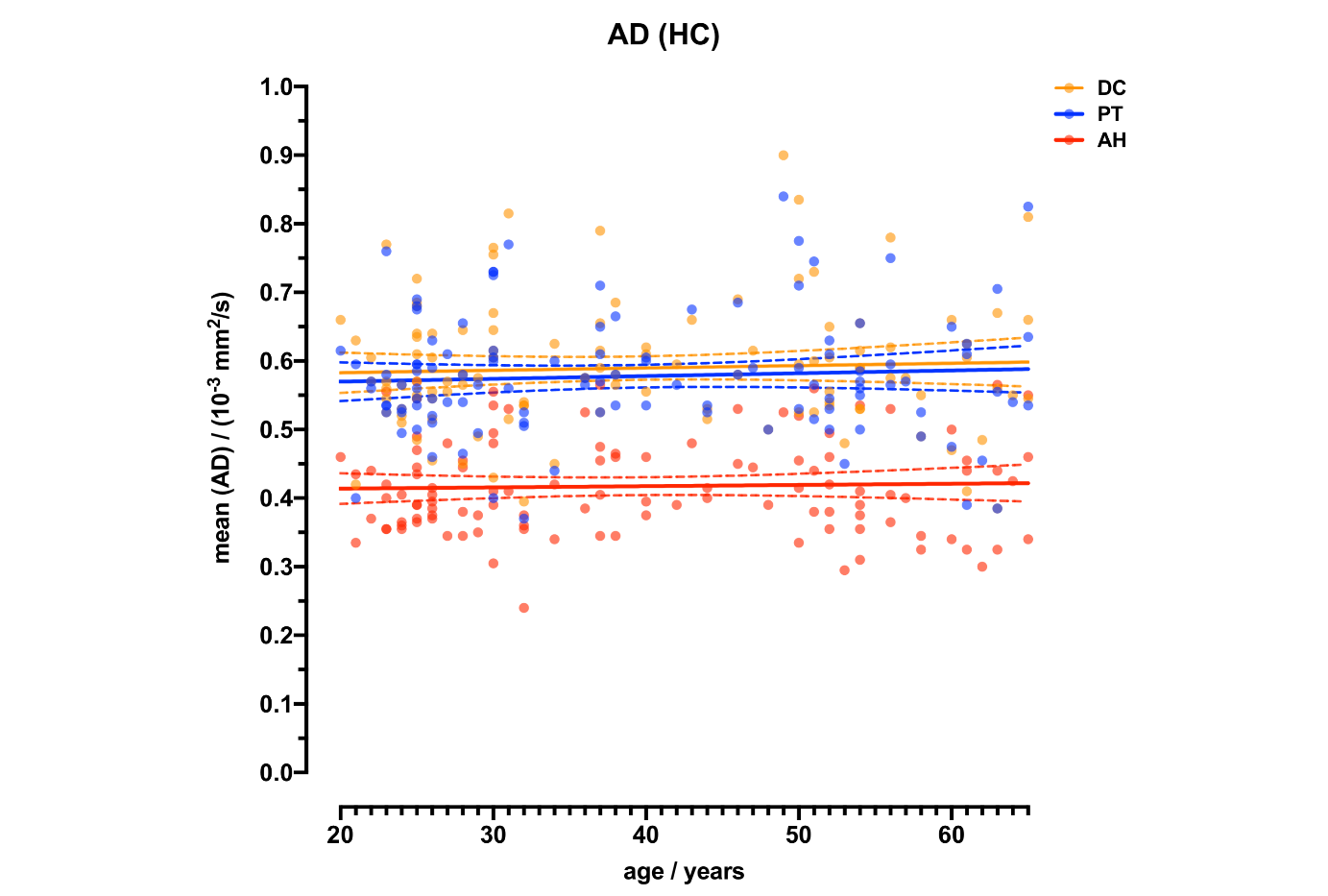


**Supplemental Figure 3: Age distribution of axial diffusivity (AD) measured in the pyramidal tracts, dorsal columns and anterior horns of healthy controls.**

|  | DTI |
| --- | --- |
| Echo Time (TE) [ms] | 1.46 |
| Time of repetition (TR) [ms] | 230 |
| Field of view (FOV) [mm] | 100x100 |
| image matrix | 128x128 |
| Voxel size [mm] | 0.78x0.78x5.0 |
| Slice thickness [mm] | 5.0 |
| Band witdh (Hz) | 752 |
| Fraction of  k-space | 6/8 |
| Parallel Imaging (iPAT/GRAPPA) | 2 |
| Number of averages | 32 |

**Supplemental Table 1:** MR sequence parameters of spinal DTI

MR examinations were performed using a 32-channel receive-only matrix head coil setup in supine position. The built-in body coil was applied for spin excitation.

| **GLM**  **HSP study patients vs. healthy controls** | | | |
| --- | --- | --- | --- |
| **parameter** | **FA** | | |
|  | **PT**  F(3, 146) = 25.809, **p = < 0.001**, R^2^ = 0.351, R^2^_adjusted_ = 0.338 | **DC**  F(3, 146) = 6.907, **p = < 0.001**, R^2^ = 0.127, R^2^_adjusted_ = 0.108 | **AH**  F(3, 146) = 4.010, p = 0.459, R^2^ = 0.078, R^2^_adjusted_ = 0.058 |
| group membership | **p = < 0.001** | **p = < 0.001** | / |
| age | p = 0.814 | p = 0.840 | / |
| sex | p = 0.182 | p = 0.973 | / |
|  | **RD** | | |
|  | **PT**  F(3, 146) = 3.751, **p = 0.012**, R^2^ = 0.073, R^2^_adjusted_ = 0.053 | **DC**  F(3, 146) = 0.424, p = 0.736, R^2^ = 0.009, R^2^_adjusted_ = - 0.012 | **AH**  F(3, 146) = 1.818, p = 0.147, R^2^ = 0.037, R^2^_adjusted_ = 0.017 |
| group membership | **p = 0.003** | / | / |
| age | p = 0.789 | / | / |
| sex | p = 0.221 | / | / |
|  | **MD** | | |
|  | **PT**  F(3, 146) = 4.010, **p = 0.009**, R^2^ = 0.078, R^2^_adjusted_ = 0.058 | **DC**  F(3, 146) = 1.084, p = 0.358, R^2^ = 0.022, R^2^_adjusted_ = 0.002 | **AH**  F(3, 146) = 1.818, p = 0.121, R^2^ = 0.037, R^2^_adjusted_ = 0.017 |
| group membership | **p = 0.007** | / | / |
| age | p = 0.645 | / | / |
| sex | p = 0.136 | / | / |

**Supplemental Table 2**: Results of univariate GLMs of HSP study patients vs. healthy controls

Univariate GLMs with age and sex as co-founding factors and subject group (HSP study patients vs. healthy controls) as fixed factor were performed. The effect of the factors were only evaluated if the GLM showed a significant in-between group difference.
